# Firing Patterns of Mitral Cells and Their Transformation in the Main Olfactory Bulb

**DOI:** 10.3390/brainsci14070678

**Published:** 2024-07-03

**Authors:** Ze-Jun Wang, Liqin Sun, Thomas Heinbockel

**Affiliations:** 1 Department of Anatomy, Howard University College of Medicine, Washington, DC 20059, USA; 2 Department of Pharmacology and Physiology, Georgetown University Medical Center, Washington, DC 20057, USA

**Keywords:** olfaction, firing pattern, GABA, glutamate, dendrodendritic, patch-clamp, brain slice, olfactory bulb, mitral cell, metabotropic glutamate receptor

## Abstract

Mitral cells (MCs) in the main olfactory bulb relay odor information to higher-order olfactory centers by encoding the information in the form of action potentials. The firing patterns of these cells are influenced by both their intrinsic properties and their synaptic connections within the neural network. However, reports on MC firing patterns have been inconsistent, and the mechanisms underlying these patterns remain unclear. Using whole-cell patch-clamp recordings in mouse brain slices, we discovered that MCs exhibit two types of integrative behavior: regular/rhythmic firing and bursts of action potentials. These firing patterns could be transformed both spontaneously and chemically. MCs with regular firing maintained their pattern even in the presence of blockers of fast synaptic transmission, indicating this was an intrinsic property. However, regular firing could be transformed into bursting by applying GABA_A_ receptor antagonists to block inhibitory synaptic transmission. Burst firing could be reverted to regular firing by blocking ionotropic glutamate receptors, rather than applying a GABA_A_ receptor agonist, indicating that ionotropic glutamatergic transmission mediated this transformation. Further experiments on long-lasting currents (LLCs), which generated burst firing, also supported this mechanism. In addition, cytoplasmic Ca^2+^ in MCs was involved in the transformation of firing patterns mediated by glutamatergic transmission. Metabotropic glutamate receptors also played a role in LLCs in MCs. These pieces of evidence indicate that odor information can be encoded on a mitral cell (MC) platform, where it can be relayed to higher-order olfactory centers through intrinsic and dendrodendritic mechanisms in MCs.

## 1. Introduction

The olfactory system must accurately identify a vast variety of odorants for the survival of mammals and insects. The mammalian main olfactory bulb (MOB) is the first site for processing odor information that is conveyed from the nasal epithelium by olfactory receptor neurons. Odor information is thought to be initially processed in glomeruli and then sent to higher-order olfactory structures via mitral/tufted cells (MCs/TCs), which form its sole output to the cortex. The human olfactory sense is often relegated to a less important role compared to vision and hearing. However, the olfactory system has emerged as a critical prodromal indicator and early biomarker of several neurodegenerative disorders, such as Parkinson’s and Alzheimer’s disease, as well as a critical factor in aging and multiple chemical sensitivity [1,2,3,4,5,6,7]. For example, in Parkinson’s disease (PD), olfactory impairment is one of the earliest and most common non-motor symptoms, and almost all PD patients are affected by olfactory deficits. Cognitive impairment and decline represent common non-motor disorders in PD patients and are often associated with hyposmia and depression and may predict the development of PD dementia [2,3,4]. Furthermore, during the COVID-19 pandemic, sudden loss of smell and taste was used as a reliable indicator of the SARS-CoV-2 disease [8,9]. As such, the human chemical senses, namely taste and smell, have seen a renaissance in terms of research, public perception, and clinical relevance.

It is known that information about external sensory stimuli, such as light, sound, taste, smell, and touch, is encoded in patterns of action potentials and transmitted in the brain [10]. Olfactory information is encoded by action potentials and network oscillations [9,10,11,12,13,14]. The timing of action potentials and the firing patterns of MCs were recognized as crucial components in the encoding of odor information [12,14,15,16]. Although the action potentials of regularly firing neurons can vary somewhat in duration, amplitude and shape, they are typically treated as identical stereotyped events in neural coding studies. In vivo, odorants evoke an excitatory response that is characterized by synaptic depolarization as a function of the respiratory cycle and on which are superimposed bursts of action potentials in MCs [17,18,19]. In slice preparations, MCs express intrinsic firing properties that are preserved when blocking synaptic transmission [20,21,22]. The intrinsic firing property of MCs in vitro and the odorant-evoked bursting of MCs in vivo imply that odor information might be encoded by modifying the firing pattern in MCs. The question is how MCs encode varying odor information with their intrinsic property of action potential firing.

Neurons in the central nervous system exhibit various activity patterns, including sting or continuous/regular firing [23,24], which are integral to olfactory processing. MCs demonstrate highly correlated responses to external odors, reflected in their average spike rates [16]. Moreover, in awake mice, MCs exhibit precise sniff-locked bursting in response to odorants within the olfactory bulb [22]. This phase-locked bursting to respiration suggests that MCs preferentially spike at specific phases of the respiratory cycle [16,25]. In response to an odor, MC firing rates can increase or decrease from resting values, and the timing of spikes in relation to respiration can be altered [16,26,27]. In addition, recent work has demonstrated that in awake animals, the heartbeat entrained the activity of a subset of olfactory bulb neurons [28]. MCs convey odor information to higher brain regions by modulation of either the number of spikes in each respiratory cycle, which is a form of a rate code, or the timing of these spikes within the cycle, which is a time code [16]. Using brain slice preparations that include the MOB, the firing patterns of MCs reported by different research group are not consistent. Our previous studies showed that MCs display spontaneous discharge, mostly as continuous firing at a modest firing frequency of 1–8 Hz [21,29], which is consistent with reports by other groups [30,31,32,33]. Meanwhile, the discharge of MCs was reported as spontaneous burst firing [21,34,35,36,37]. Even though the characterization of the two distinct firing patterns in the primary output neurons is a fundamental component to understand the odor information processing in MOB, the relationship between these two different firing patters and their underlying mechanisms remains unclear. 

Brain slice experiments suggest that activation of the olfactory nerve leads to highly correlated activation of MCs [16,38,39]. Synchronization of MCs, evoked by electrical stimulation of afferents, is glomerulus-specific and requires release of glutamate among dendrites of MCs [40]. The coupling between the primary dendrites of MCs is thought to occur through gap junctions [37,41] or glutamate spillover [42,43]. These findings imply that the MC spike rate and even firing pattern may be strongly influenced by synaptic or dendrodendritic connection of neurons. Here, we hypothesize that the two types of firing patterns of MCs can be transformed under certain condition through intrinsic and dendrodendritic mechanisms.

Using whole-cell patch-clamp recording in a slice preparation of the mouse MOB, we present evidence to illustrate how neurotransmitters regulate intrinsic properties of neurons, shape firing patterns of neurons, and reveal the underlying mechanisms in the MOB.

## 2. Materials and Methods

### 2.1. Animals and Slice Preparation

Wild-type mice (C57BL/6J, Jackson Laboratory, Bar Harbor, ME, USA) were used in agreement with Institutional Animal Care and Use Committee and NIH guidelines. Juvenile (16–25 day old) mice were decapitated, and the MOBs were dissected out and immersed in artificial cerebrospinal fluid (ACSF, see below) at 4 °C, as previously described [21]. Horizontal slices (400 µm thick) were cut parallel to the long axis using a vibratome (Vibratome Series 1000, Ted Pella Inc., Redding, CA, USA). For recording, a brain slice was placed in a recording chamber mounted on a microscope stage and maintained at 30 ± 0.5 °C by superfusion with oxygenated ACSF flowing at 2.0–2.5 mL/min.

### 2.2. Electrophysiology

Visually guided recordings were obtained from cells in the MCs/TCs layer and glomerular layer with near-infra red differential interference contrast optics and a BX51WI microscope (Olympus Optical, Tokyo, Japan) equipped with a camera (C2400-07, Hamamatsu Photonics, Hamamatsu, Japan). Images were displayed on a Sony Trinitron Color Video monitor (PVM-1353MD, Sony Corp., Tokyo, Japan). Recording pipettes (5–8 MΩ were pulled on a Flaming-Brown P-97 puller (Sutter Instrument Co., Novato, CA, USA) with 1.5 mm O.D. borosilicate glass with filament. Seal resistance was routinely >1 GΩ, and liquid junction potential was 9–10 mV; reported measurements were not corrected for this potential. Data were obtained using a Multiclamp 700B amplifier (Molecular Devices, Sunnyvale, CA, USA). Signals were low-pass Bessel filtered at 2 kHz and digitized on computer disc (Clampex 10.1, Molecular Devices). Data were also collected through a Digidata 1440A Interface (Molecular Devices) and digitized at 10 kHz. Holding currents were generated under control of the Multiclamp 700B Commander. Numerical data were expressed as the mean ± SEM. Tests for statistical significance (*p* < 0.05) were performed using a paired Student’s *t*-test or one-way ANOVA followed by the Bonferroni test for multiple comparisons. Membrane potentials were calculated from the steady-state membrane potential that occurred after an action potential (resting potential) during control or drug treatment, respectively. The burst membrane potential was measured as the platform of depolarization. The afterhyperpolarization (AHP) was measured as the most negative value of membrane potential following the depolarizing burst, and its peak usually occurred within 400 ms after the end of the burst.

The artificial cerebrospinal fluid (ACSF) consisted of (in mM): NaCl (124), KCl (3), CaCl_2_ (2), MgSO_4_ (1.3), glucose (10), sucrose (4.4), NaHCO_3_ (26), and NaH_2_PO_4_ (1.25) (pH 7.4, 300 mOsm), saturated with 95 O_2_/5% CO_2_ (modified from [20]). For intracellular recording of spiking activity, the pipette-filling solution consisted of (mM) K-gluconate (144), MgCl_2_ (2), HEPES (10), Mg_2_ATP (2), Na_3_GTP (0.2), NaCl (2), and EGTA (0.2) (pH 7.3, 290 mOsm).

The following drugs were bath applied: L-2-amino-5-phosphonopentanoic acid (AP5, APV, a ionotropic glutamate receptor antagonist), 6-cyano-7-nitroquinoxaline-2-3-dione (CNQX, a ionotropic glutamate receptor antagonist), (2-(3-carboxypropyl)-3-amino-6-(4 methoxyphenyl)-pyridazinium bromide (gabazine, SR-95531; a GABAergic receptor antagonists), and picrotoxin (a GABAergic receptor antagonist). Chemicals and drugs were supplied by Sigma-Aldrich (St. Louis, MO, USA) and Tocris (Ellisville, MO, USA).

## 3. Results

Recordings were obtained from 697 MCs in mouse MOB slices. MCs were identified visually by their soma location and relatively large soma size, and by their input resistance (284 ± 16.6 MΩ, *n* = 69). The membrane potential of MCs in this study was −50.5 ± 0.6 mV (*n* = 69). In whole-cell current-clamp recording mode, MCs spontaneously generated two distinct firing patterns under our recording condition: (1) regular firing, i.e., regular discharge of single action potentials (*n* = 444), and (2) burst firing, i.e., discharge of bursts of action potentials (*n* = 253). A total of 35 out of 444 regular firing MCs (less than 8%) spontaneously transformed their firing pattern into burst firing. In slices, the MC spontaneous, regular discharge is more modest (1–8 Hz; mean: 3.2 Hz, *n* = 10), and the burst rate is at 0.04–0.45 burst/sec with an intra-burst firing frequency at 9–35 Hz (mean: 14. 5 Hz, *n* = 9).

### 3.1. Blockade of GABAergic Synaptic Transmission Alters Both Regular Firing and Burst Firing of Mitral Cells

In slices, most MCs (~60.2%) displayed spontaneous regular discharge of action potentials. Approximately 34.6% of MCs displayed burst firing with periods of rapid firing followed by quiescent, silent periods. About 5.2% of MCs spontaneously transformed their firing patterns between regular and burst firing, mostly switching from a regular to a burst firing pattern.

It has been reported that MC spontaneous firing is intrinsically generated and persists in the presence of blockers of ionotropic glutamate and GABA receptor antagonists [20,44]. The persistence of the firing pattern of MCs/TCs in the presence of ionotropic glutamate and GABA receptor blockers has been interpreted such that it is based on intrinsic membrane properties because the firing is generated by the cell itself without the involvement of synaptic transmission. As mentioned above, we observed two distinct firing patterns in MCs that were recorded in the same brain slice, suggesting that the MOB may have an intrinsic mechanism that allows the firing pattern to be transformable. We hypothesize that spontaneous synaptic transmission may be involved in the intrinsic mechanism that makes the firing pattern transformable.

**Figure 1:**
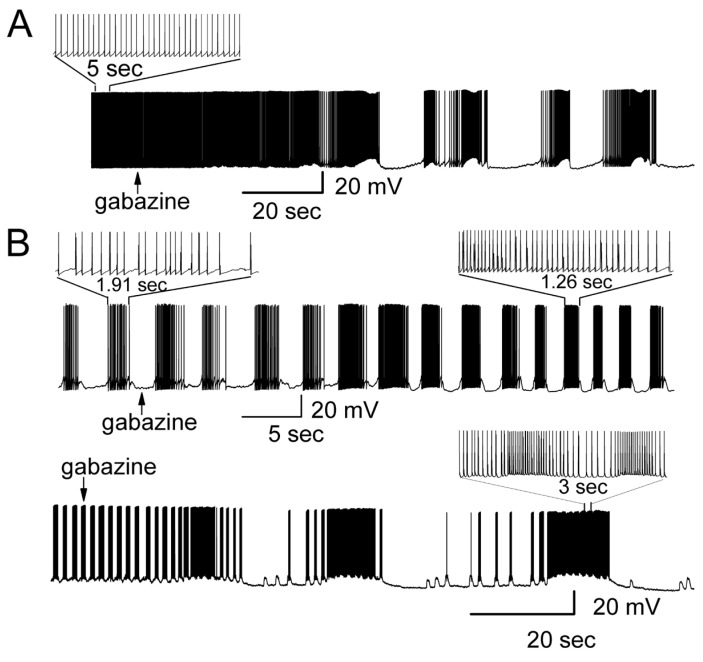
Blocking GABAergic (inhibitory) transmission dramatically modulated both spontaneous regular firing and burst firing patterns of MCs. (**A**) Gabazine (5 μM) altered the spontaneous regular firing pattern. Current-clamp whole-cell recordings were made from an MC exhibiting regular firing that was transformed to burst firing. Expanded traces showed the regular firing. (**B**) Gabazine enhanced the strength of burst firing. Upper recording: gabazine-modified firing pattern increased the number of spikes per burst and MMP. Lower recording: gabazine modified firing pattern with greatly increased strength of bursting that contained sub-bursts of firing. Expanded traces showed sub-bursts of firing.

To test this hypothesis, we blocked inhibitory GABAergic transmission, demonstrated as spontaneous inhibitory postsynaptic currents (IPSC) in the MCs of MOB slices [45], by bath application of ionotropic γ-aminobutyric acid (GABA) receptor antagonists to determine if the firing properties are modulated. We found that both GABAergic receptor antagonists (gabazine and picrotoxin) facilitated the switch of the most regular firing pattern to burst firing. In MOB slices, the switch from a regular firing pattern to rhythmic burst firing was observed in 27 out of 30 cells in response to bath application of gabazine (5 μM) (Figure 1A). In MCs with regular firing, gabazine evoked a membrane depolarization with drastically increased frequency of action potentials. This was followed by membrane hyperpolarization resulting from the activation of potassium channels. Subsequently, the cell repolarized to resting membrane potential and then repeated the same cycle of depolarization and hyperpolarization, forming the rhythmic burst firing (Figure 1A). The evoked bursting activity exhibited varying membrane potential in MCs. In order to assess the gabazine effect on the membrane potential, the membrane depolarization was determined as (a) the intra-burst membrane potential occurring during the depolarization platform of bursting, and (b) the minimal membrane potential (MMP) occurring at the end of each burst and depolarization platform [46]. In control conditions, MCs exhibited a background action potential firing rate ranging from 1 to 8 Hz (*n* = 69). In the presence of gabazine, the occurrence of burst firing typically started with a varying duration of depolarization (duration of bursting: 0.2 to 3 s; depolarization: 0.8 to 5 mV, averaged difference between depolarization potential and membrane potential: ΔVm = 2.1 ± 0.5 mV, *n* = 7, *p* = 0.02), followed by bursting with an intra-burst frequency of 4.6 to 30 Hz (average: 13.9 ± 1.8 Hz, *n* = 8), and hyperpolarization (membrane potential in control vs. MMP: ΔVm = −3.5 ± 0.6 mV, *n* = 7, *p* = 0.006).

The influence of gabazine on the spontaneous bursting properties of MCs was also examined (Figure 1B). In particular, we tested whether the strength of bursting (evaluated as the number of spikes per burst, intra-burst frequency) could be modulated by blockade GABA_A_ receptors. We also quantified several other parameters, such as burst duration, burst membrane potential, the frequencies of bursts, and MMP [47]. Blocking fast inhibitory synaptic transmission and GABA_A_ receptors by gabazine altered the bursting pattern by increasing the strength of bursting, enhancing the bursting membrane potential, hyperpolarizing MMP, and shortening the duration of bursting (Figure 1B, upper recording). In some MCs, gabazine greatly increased the strength of bursting with some sub-bursts of firing included in the main bursting (Figure 1B, lower recording). Compared to the MCs with spontaneous bursting, bath application of gabazine significantly increased the intra-burst frequency from 14.5 ± 2.6 Hz to 46.5 ± 6.8 Hz (*n* = 7, *p* = 0.019), and hyperpolarized MMP (membrane potential vs. MMP) from −1.7 ± 0.4 mV to −6.1 ± 1.0 mV (*n* = 7, *p* = 0.0071). Gabazine changed the burst duration (control: 2.1 ± 0.35 s; in gabazine: 1.68 ± 0.32 s, *n* = 7, *p* = 0.036, paired *t*-test), increased the burst membrane potential (ΔVm = 1.4 ± 0.2 mV, *n* = 7, *p* = 0.031), and enhanced the inter-burst frequency (control: 0.3 ± 0.2 bursts/s, blockers: 0.4 ± 0.2 bursts/s, *n* = 8, *p* = 0.021, paired *t*-test). These results demonstrated that GABA_A_ receptor antagonist dramatically altered both spontaneous regular and burst firing pattern of MCs.

**Figure 2:**
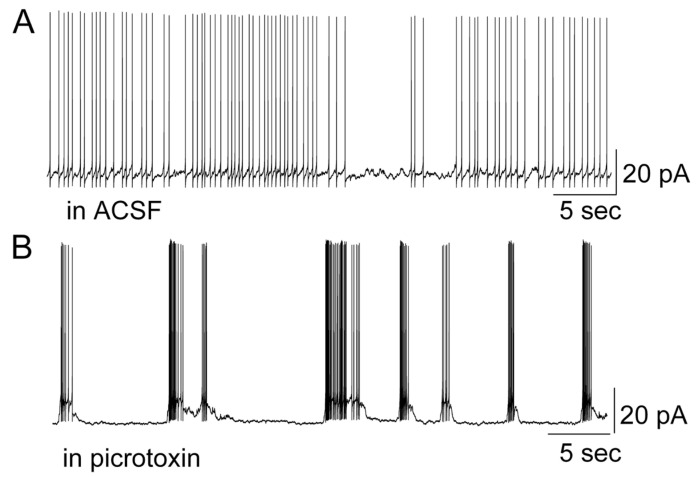
Picrotoxin modulated the firing pattern in a mitral cell. (**A**) Regular firing of action potentials in a mitral cell. (**B**) Picrotoxin-evoked burst firing in the same mitral cell.

Similarly, another GABA_A_ receptor antagonist, picrotoxin, also demonstrated an effect on firing pattern transformation (Figure 2) (*n* = 4). Picrotoxin had a similar capability of driving a switch from the spontaneous regular firing pattern to the bursting pattern.

### 3.2. The Effects of GABA_A_ Receptor Antagonists on Firing Pattern Can Be Reversed by Blocking Glutamatergic Synaptic Transmission

The above results indicated that GABA_A_ receptor antagonists dramatically transformed regular firing to burst firing or enhanced the strength of MC spontaneous bursting. The evoked rhythmic burst firing of MCs suggests that MCs generated action potentials synchronically. Previous work from other labs indicated that synchronization of long-lasting depolarizations (LLDs) was generated only in MCs whose apical dendrites ramify in the same glomerulus and medicated by a spillover of glutamate release [34,37,40,48]. Because the burst firing described above displayed similar frequencies to that of LLDs, the transformation of the firing pattern by gabazine may share similar mechanisms underlying them. Therefore, we tested if evoked burst firing could be reversed by blocking glutamatergic transmission.

**Figure 3:**
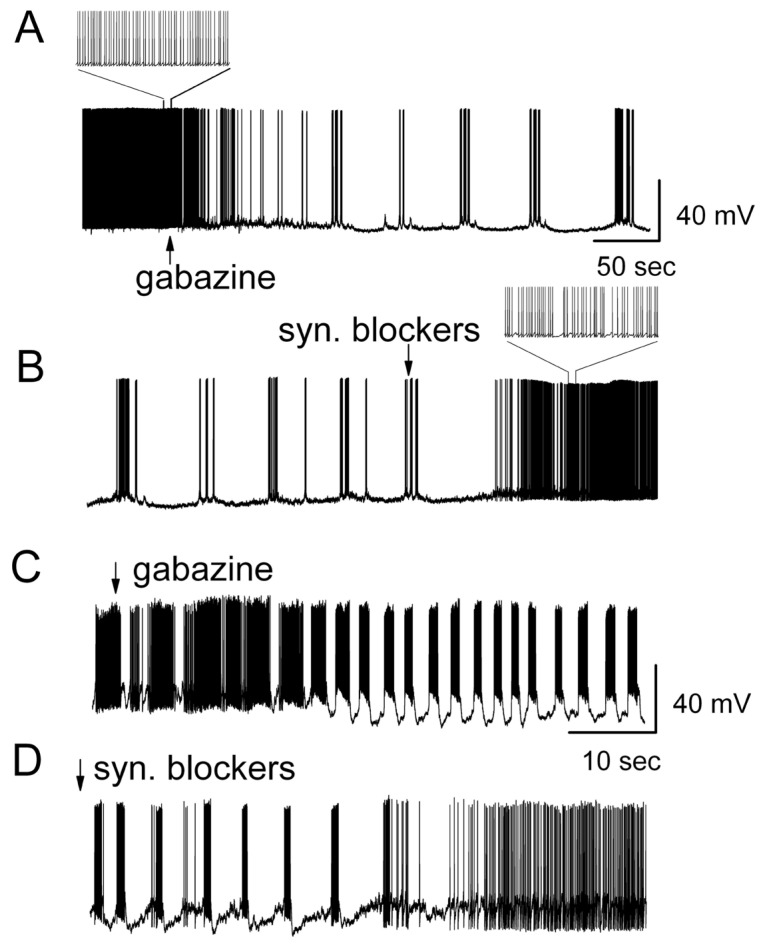
Ionotropic glutamate receptor antagonists reversed the gabazine-evoked burst firing in mitral cells. (**A**) Gabazine drove a change in the firing pattern from regular single spikes to burst firing. (**B**) In the presence of gabazine, additional application of CNQX (10 μM) plus D-AP5 (50 μM) reversed the burst firing to regular firing. ((**A**,**B**) are from the same representative mitral cell). Synaptic blockers included ionotropic glutamate and GABA_A_ receptor blockers (CNQX, 5-AP and gabazine). (**C**,**D**) were from the same mitral cell that showed burst firing. Gabazine enhanced the strength of bursts (**C**) and additional bath application of CNQX plus D-AP5 significantly reversed the firing pattern back to regular single spikes (**D**).

**Figure 4:**
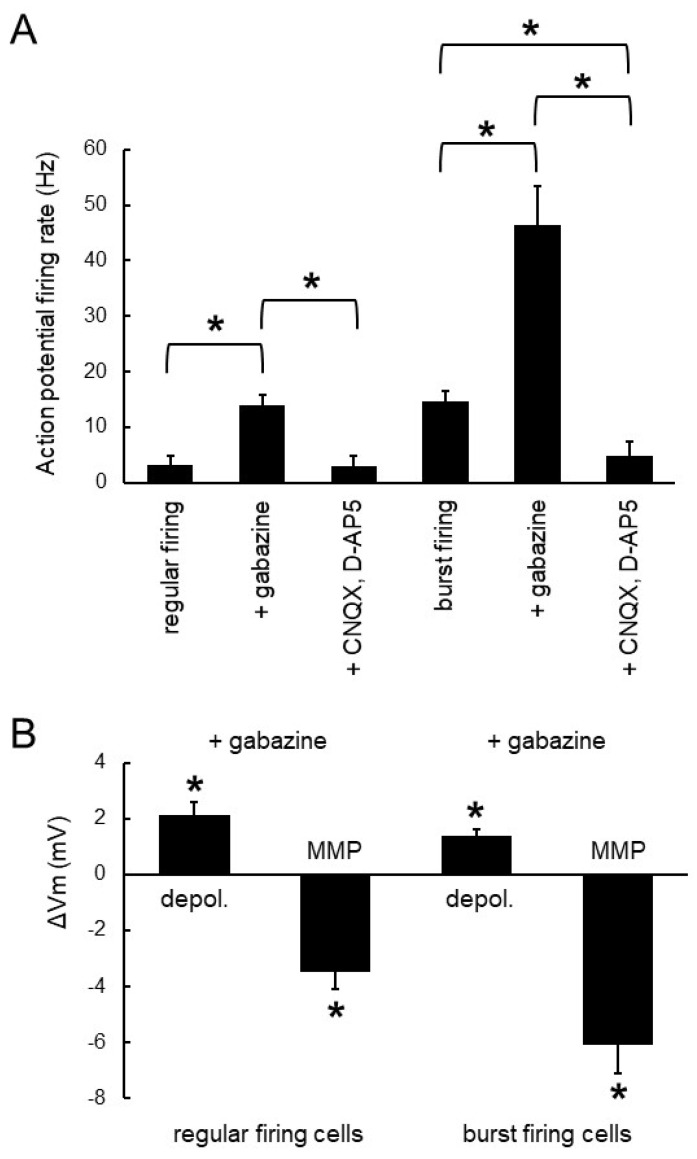
Quantitative analysis of gabazine-evoked burst firing in mitral cells and its reversal by ionotropic glutamate receptor antagonists. (**A**) Gabazine increased the firing frequency in regular firing cells and burst firing cells. The increase was reversed to regular firing by additional application of ionotropic glutamate receptor antagonists CNQX plus D-AP5. (**B**) Gabazine depolarized the membrane potential (depol.) in regular firing cells and burst firing cells during the intra-burst depolarization platform. Gabazine hyperpolarized cells after each burst measured as the minimal membrane potential (MMP) occurring at the end of the depolarization platform of each burst. ‘*’ indicates significance (*p* < 0.05).

Figure 3 demonstrates that ionotropic glutamate receptor antagonists (CNQX, plus D-AP5) reversed the ionotropic GABA_A_ receptor antagonist-evoked burst firing. Bath application of gabazine transformed the firing pattern from spontaneous regular firing into bursting (Figure 3A). In the presence of gabazine, additional application of CNQX (10 μM) plus D-AP5 (50 μM), as synaptic blockers, significantly reversed the burst firing into regular single spikes (Figure 3B). This suggested that ionotropic glutamate receptors were involved in the generation of the firing transformation. All of the cells tested that transformed their firing pattern in response to gabazine were subsequently able to be reversed to their previous single spike firing by application of ionotropic glutamate receptor antagonists (*n* = 7). In MCs with spontaneous burst firing, gabazine enhanced the strength of bursts (Figure 3C). Additional bath application of CNQX plus D-AP5 significantly switched the burst firing pattern in these MCs to firing with more single spikes (Figure 3D, *n* = 6). Figure 4 show the quantitative analysis of overall gabazine-evoked burst firing in MCs and its reversal by ionotropic glutamate receptor antagonists.

In summary, the burst firing evoked by gabazine in regular firing MCs and the spontaneous burst firing pattern of MCs were reversibly transformed to regular single spike firing or less burst firing by blocking ionotropic glutamate receptors. This suggested that the burst firing pattern of MCs was mediated by ionotropic glutamatergic transmission. Furthermore, the results suggested that the regular firing in MCs was intrinsically mediated, whereas the burst firing was a phenomenon based on synaptic transmission.

### 3.3. Activation of GABA Receptors Inhibited Burst Firing by Tonically Silencing Mitral Cells, Rather than Reversing Burst Firing into Regular Firing

The results in Figure 3 showed that a blockade of ionotropic glutamatergic transmission could eliminate the GABA_A_ receptor antagonists-evoked modification of the MC spontaneous firing pattern. To determine the role the GABA_A_ receptors in the modification of MC firing patterns, we tested the effects of GABA on MC firing patterns.

**Figure 5:**
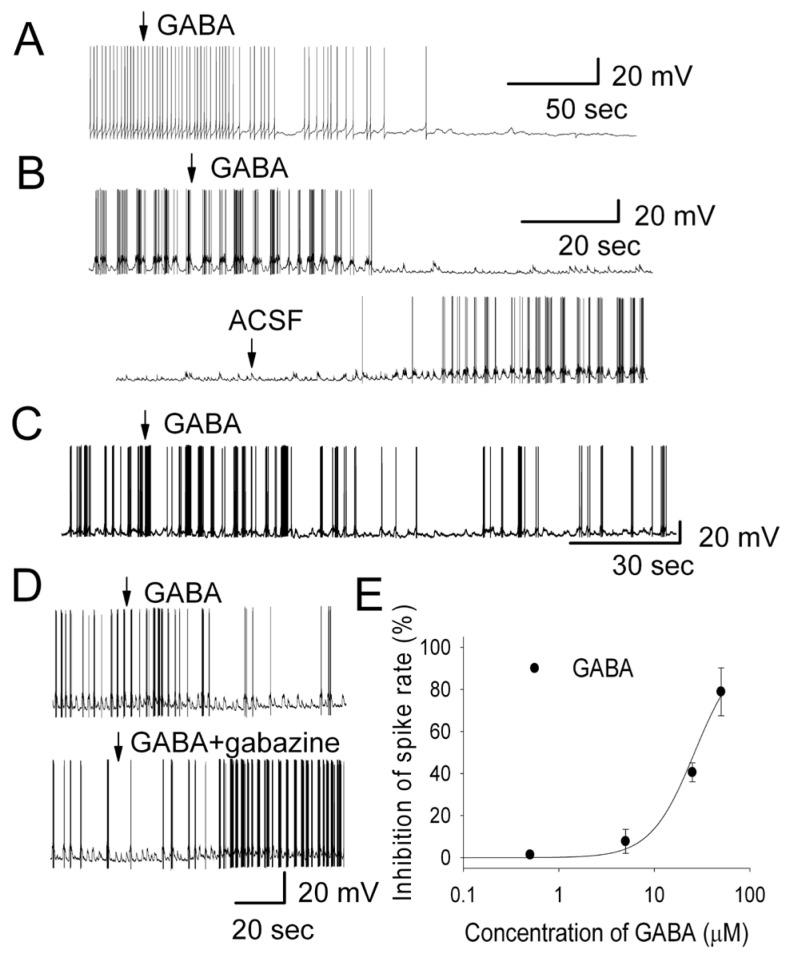
GABA receptor agonist inhibited both regular and burst firing. (**A**) GABA (50 μM) tonically inhibited regular firing and hyperpolarized the cell. (**B**) GABA (50 μM) reversibly inhibited burst firing of a MC and hyperpolarized the cell. (**C**) GABA inhibited the strength of burst firing but did not switch the firing pattern from burst firing to regular firing. (**D**) In a burst firing MC, gabazine reversed the GABA-elicited inhibition of firing activity. (**E**) Concentration–response curve of GABA-evoked inhibition on MC activity. The GABA-evoked change in spiking rate was normalized to the control condition, and then averaged. Each point was the mean value ± S.E.M. of seven cells. The line is fit for the data to the Hill equation: *y = Y*_0_ + *Ax^n^*/(*K_a_^n^* + *x^n^*), where *y* is the inhibition of spiking rate, *x* is concentration of drugs, *Y*_0_ is minimal inhibition, *A* is maximal inhibition, *Kd* is the apparent dissociation constant for agents, and *n* is the Hill coefficient. *Kd* and *n* were estimated using a Marquardt nonlinear least-squares routine.

In MCs with regular firing, GABA drastically reduced the frequency of action potentials and hyperpolarized the cell, but it did not alter the pattern of regular firing (Figure 5A). In MCs with spontaneous burst firing, GABA reversibly inhibited the bursting but preserved the pattern of burst firing, as seen as small membrane depolarizations (Figure 5B). At a concentration of 50 μM of GABA, the overall firing rate of both regular and burst firing was dramatically reduced from 5.0 ± 0.8 Hz to 1.7 ± 0.3 Hz (*n* = 6; *p* < 0.001; paired *t* test). The reduction in the firing rate was accompanied by hyperpolarization of the MC membrane potential by −1.1 ± 0.3 mV (*n* = 6; *p* < 0.05; paired *t* test). The potent tonic inhibition by GABA is consistent with previous reports showing that GABA receptors are abundant in MCs [49,50].

Figure 5C demonstrates that activation of GABA_A_ receptors only inhibited the strength of bursting rather than transforming the firing pattern into regular firing, indicating that gabazine-elicited switches of the firing pattern were not mediated by GABA_A_ receptors. The GABA-evoked inhibition of firing activity was reversed by gabazine under the same condition (Figure 5D) (*n* = 10), suggesting that GABA and gabazine competitively bind to GABA_A_ receptors to regulate the excitability of MCs. Figure 5E demonstrates the concentration–response curve of GABA on MC firing rate at varying concentrations (*n* = 4~10). The averaged inhibitory effects evoked by varying concentrations of the GABA were well fit by the Hill equation and allowed us to estimate the EC_50_ of 28.8 μM.

The above results indicated that a GABA_A_ receptor agonist neither altered the firing pattern nor reversed the burst firing pattern into regular firing, but rather inhibited the strength of firing of both spontaneous regular firing and burst firing. This indicated that the generation of a burst firing pattern by blocking GABA_A_ receptors was not mediated through GABAergic transmission and GABA_A_ receptors. The results suggested that the disinhibition of the cell through blockade of GABA_A_ receptors probably activated other neurotransmitter systems, e.g., the glutamatergic synaptic transmission.

### 3.4. Ionotropic Glutamate Receptor Antagonists Blocked the Firing Pattern Transformation from Regular to Burst Firing

**Figure 6:**
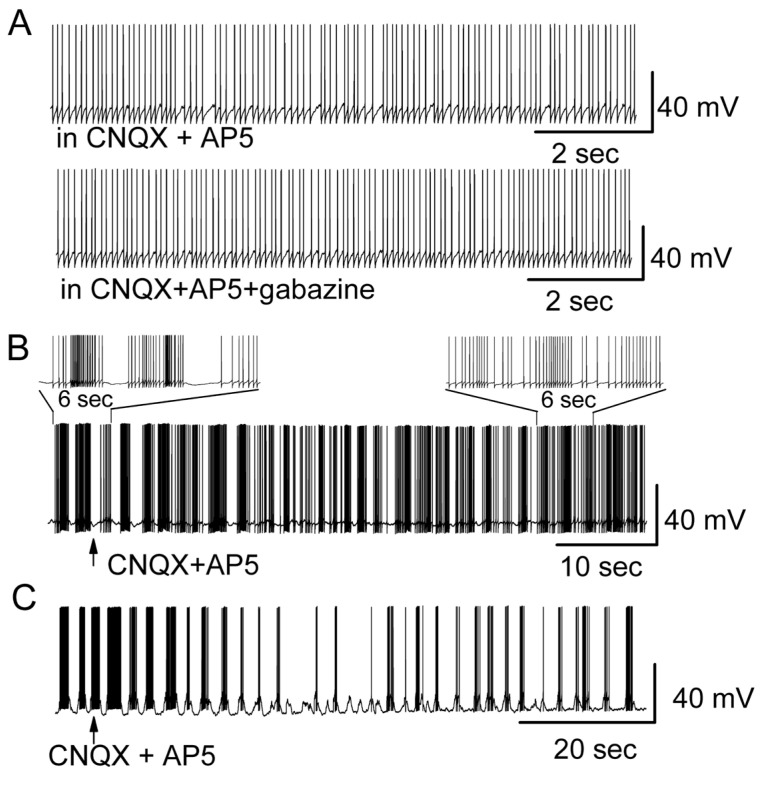
Ionotropic glutamate receptor antagonists blocked the transformation of the firing pattern from regular to bursts or modulated the burst firing pattern to display less bursting. (**A**) In the presence of CNQX plus AP5, additional bath application of gabazine failed to evoke burst firing in a regular firing MC. (**B**) CNQX plus AP5 transformed the spontaneous burst firing of a MC to much more regular firing or (**C**) reduced the strength of bursting in an over-excited MC which was pretreated with gabazine for more than 20 min. (**B**,**C**) were from different MCs.

Blockers of fast synaptic transmission (NMDA and AMPA receptor antagonists, AP5 and CNQX) are commonly used to block synaptic transmission in many experimental conditions [22]. It is thought that MC spontaneous firing is intrinsically generated because it persists in the presence of fast synaptic blockers [20,44]. Our results partly supported the idea that only spontaneous regular firing persisted in the presence of fast synaptic blockers (Figure 6A). Fast synaptic blockers did not significantly change the regular firing pattern, firing rate and membrane potential. Compared to that without fast synaptic blockers, the firing rate with fast synaptic blockers was 109.1 ± 10.0% (*n* = 20, *p* = 0.36, paired *t* test), and the difference in membrane depolarization (ΔVm) was 0.3 ± 0.1 mV (*n* = 20, *p* = 0.09, paired *t* test). However, in spontaneously burst firing MCs, the blockers obviously transformed the burst firing pattern into much more regular pattern (*n* = 7) (Figure 3B,D).

The question arose as to why the spontaneous burst firing pattern rather than the regular firing pattern of MCs was transformed while synaptic transmission was blocked. To determine the mechanisms underlying the alteration of firing pattern, we tested the role of ionotropic glutamatergic transmission in this regard. Bath application of ionotropic glutamate receptor antagonists AP5 plus CNQX to regular firing MCs failed to change their firing pattern (Figure 6A, above) (*n* = 11). In the presence of AP5 plus CNQX, additional bath application of gabazine failed to drive the switch of the firing pattern from single spikes to bursts (Figure 6A, below) (*n* = 8), which contrasted with the effect of gabazine when it was applied alone (Figure 1). This result indicated that ionotropic glutamatergic transmission was involved in the alteration of the firing pattern. By blocking ionotropic glutamatergic transmission, the change in firing pattern evoked by gabazine was prevented.

To determine if the modulation of the firing pattern by synaptic blockers was indeed mediated by glutamate receptors rather than GABA_A_ receptors, the effect of glutamate receptor antagonists was also tested on the spontaneous burst firing pattern of MCs. By applying AP5 plus CNQX to the bathing solution, while recording from spontaneously burst firing MCs, the burst pattern was changed to either much more regular firing (Figure 6B) (*n* = 7) or a reduced strength of bursting (Figure 6C). The number of spikes per burst (intra-burst frequency) was reduced from 12.8 ± 3.1 Hz to 5.0 ± 1.2 Hz (*n* = 6, *p* = 0.008), and the MMP depolarized by 1.6 ± 0.3 Hz (*n* = 6, *p* = 0.0024). The results indicated that spontaneous burst firing of MCs involved ionotropic glutamatergic transmission.

### 3.5. Gabazine Evoked or Enhanced Long-Lasting Inward Currents That Were Blocked by Ionotropic Glutamate Receptor Antagonists

To further explore the mechanism(s) underlying the change in firing pattern evoked by GABA receptor antagonists in MCs, the GABA receptor antagonist-evoked modulation of ionic currents was examined.

**Figure 7:**
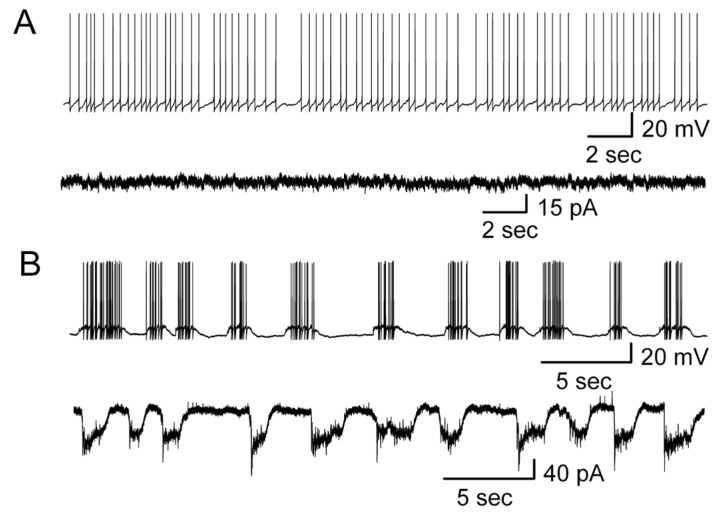
Correlation of firing pattern and long-lasting currents. (**A**) A cell with regular firing in current-clamp recording mode (above) displayed a flat line in voltage clamp recording mode (below), i.e., no significant inward current was observed. HP = −60 mV. (**B**) Burst firing recorded in current-clamp mode (above) was expressed as long-lasting inward currents recorded in voltage-clamp mode (below). HP = −60 mV.

In voltage-clamp recording mode with a holding potential at −60 mV, the cells with regular firing displayed a flat line, suggesting that regular firing of MCs did not elicit significant inward currents (Figure 7A). In contrast, in cells with spontaneous burst firing or burst firing induced by gabazine, the bursts corresponded to recurrent long-lasting depolarizing inward currents (LLCs) (Figure 7B).

The above results indicated that only burst firing evoked LLCs in MCs (Figure 7). Therefore, the mechanism underlying the change in firing pattern may be strongly related to the generation of long-lasting depolarizations (LLDs). Carlson et al. [48] demonstrated that MCs in the adult rat olfactory bulb express LLDs that occur spontaneously, in response to olfactory nerve stimulation, and after antidromic activation of M/T cells. The generation of LLDs involves recurrent, intraglomerular dendrodendritic interactions among M/T cells with a spillover of the released glutamate [37,40,48].

**Figure 8:**
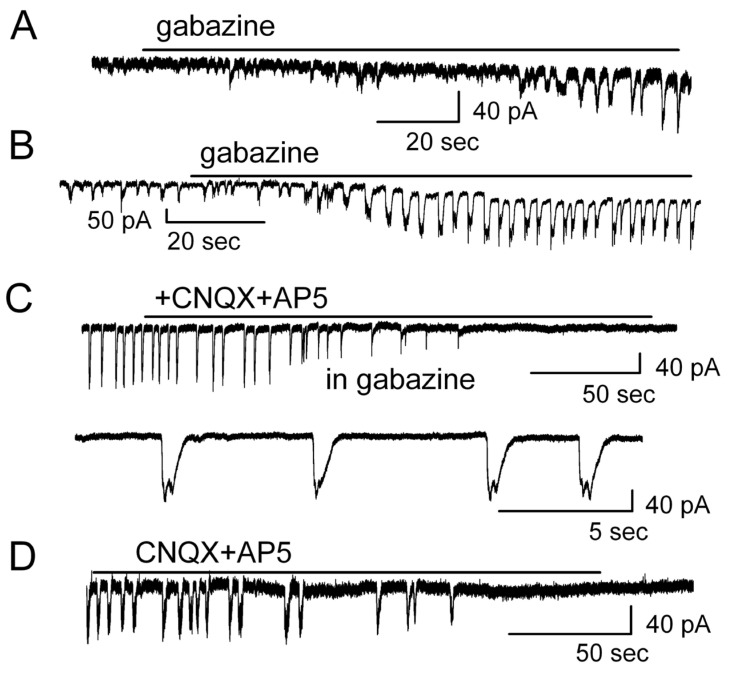
Ionotropic glutamate receptor antagonists blocked the long-lasting inward currents. (**A**) Gabazine (5 μM) induced LLCs in MC with regular firing. (**B**) Gabazine (5 μM) enhanced (**B**) LLCs in MC with spontaneous burst firing. Both induced and enhanced LLCs were blocked by CNQX plus AP5 (**C**). Below: larger time scale from (**C**). (**D**) Ionotropic glutamate receptor antagonists blocked spontaneous LLCs.

As the next step, mechanisms of generation of spontaneous LLCs, gabazine-evoked or enhanced LLCs, and their blockade by ionotropic glutamate receptor antagonists were examined. As shown above, gabazine evoked a change in firing pattern toward burst firing (Figure 1). Correspondingly, blockade of GABA receptors induced or enhanced LLCs (Figure 8). Figure 8A shows that blocking GABA_A_ receptors with gabazine elicited LLCs, starting with varying duration of slow inward currents in a regular firing MC cell. The averaged amplitude of evoked inward currents was 138.8 ± 15.8 pA (*n* = 6) with an LLC frequency of 0.3 ± 0.1 Hz (*n* = 6). In these cells, gabazine gradually induced a depolarizing inward current and evoked LLCs. This indicated that the generation of LLCs required the initiation of lasting depolarizations of MCs. LLCs also occur spontaneously in MCs with burst firing (*n* = 39). Gabazine significantly increased the amplitude and frequency of spontaneous LLCs (Figure 8B). The averaged amplitude of LLCs was enhanced from 75.8 ± 16.0 pA to 153.6 ± 14.9 pA (*n* = 13, *p* < 0.001). The LLC frequency was increased from 0.17 ± 0.04 Hz to 0.25 ± 0.04 Hz (*n* = 13, *p* < 0.001). Gabazine not only evoked LLCs but also elicited a sustained depolarizing inward current of 39.5 ± 10.7 pA (*n* = 12, *p* < 0.005). The magnitude of the inward current was determined by comparing baseline currents before and 2 min after gabazine addition. The frequency and amplitude of LLCs elicited by gabazine in cells with and without spontaneous LLCs were similar (*p* > 0.05 determined by ANOVA and Bonferroni post hoc analysis; frequency: *p* = 0.40; amplitude: *p* = 0.77), suggesting that the generation of the two LLCs shares the same mechanism(s).

Similarly, the blockade of the gabazine-induced (Figure 8A), gabazine-enhanced (Figure 8B), or spontaneous LLCs (Figure 8D) by ionotropic glutamate receptor antagonists CNQX plus AP5 (Figure 8C,D) suggest that ionotropic glutamatergic transmission are involved in the generation of LLCs. The results also supported the hypothesis that spontaneous LLCs and gabazine-evoked LLCs shared the same mechanism of LLC initiation and development.

The above results suggest the involvement of a dendrodendritic mechanism in the change in firing pattern. It is commonly accepted that the spiking and firing pattern encode odor information. Thus, both intrinsic and dendrodendritic mechanisms were involved in the development of the firing patterns of MCs. Coding of odor information may be carried out on an MC platform in which intrinsic regular firing can be transformed into burst firing through dendrodendritic mechanisms.

### 3.6. The Involvement of mGluRs in LLCs in Mitral Cells

**Figure 9:**
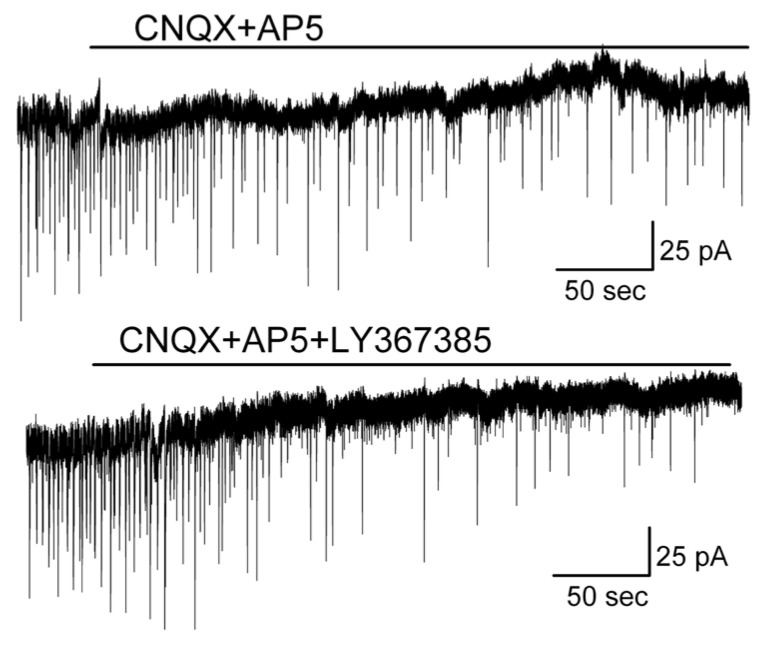
Ionotropic glutamate receptor antagonists failed to completely block LLCs in mitral cells which had been treated with gabazine for more than 20 min. Top trace: CNQX plus AP5 did not completely block LLCs in a strongly activated mitral cell. Lower trace: addition of metabotropic glutamate receptor antagonist LY367385 (20 μM) greatly improved the inhibition of LLCs in the same mitral cell.

**Figure 10:**
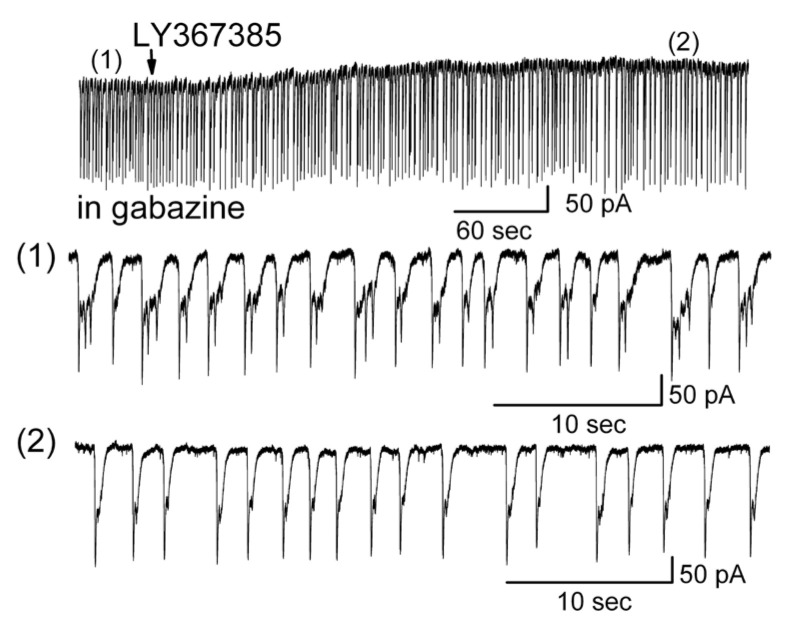
An mGluR antagonist shortened the LLC duration in MCs during application of gabazine. The upper trace shows the original recording from a mitral cell during LY367385 (20 μM) application in the presence of the GABA_A_ receptor antagonist gabazine. The upper trace is shown at an extended time scale in the middle (1) and lower traces (2). The middle trace (1) shows the LLCs in the presence of gabazine, while the lower trace (2) shows the LLCs after application of LY367385.

The above results show that ionotropic glutamate mediated the transformation of firing patterns (Figure 3) and that the spontaneous or evoked LLCs could be blocked byionotropic glutamate receptor antagonists (Figure 8). However, in certain experimental conditions, such as long-term treatment of MCs with gabazine or K^+^ channel blocker 4-AP for a longer time, e.g., more than 20 min, ionotropic glutamate receptor antagonists failed to completely block LLCs in MCs. Long-term treatment (≥20 min) with gabazine (*n* = 5) or 4-AP (*n* = 3) resulted in the failure of a complete blockade of LLCs by CNQX + AP5. However, the inhibitory effect of ionotropic glutamate receptor blockers on LLCs could be greatly improved by adding LY367385, a metabotropic glutamate receptor (mGluR) antagonist (Figure 9). This suggested that metabotropic glutamate receptors were involved in the development of LLCs in conditions where LLCs were persistently activated in MCs. The failure of CNQX + AP5 to completely block LLCs was consistent with our result that ionotropic glutamate receptor antagonists inhibited the strength of strong bursting but did not fully reverse a burst firing pattern into a regular firing pattern (shown in Figure 6C). The involvement of mGluRs was further supported by directly measuring the dynamic influence of LY36785 on LLCs in MCs which were treated with gabazine for an extended time (20 min). Figure 10 shows that in the presence of gabazine, LY36785 shortened the duration of LLCs from 1.14 ± 0.16 s to 0.58 ± 0.07 s (trace (1) vs. trace (2), *n* = 5, *p* = 0.017, paired *t*-test) without significantly changing the amplitude of the LLCs. These results suggest that strongly activated MCs could, in turn, activate mGluRs and lead to the failure of LLC blockade by ionotropic glutamate receptor antagonists alone. Similarly, enhanced LLCs evoked by a long treatment (20 min) with the K^+^ channel blocker 4-AP were not completely blocked by CNQX + AP5 (*n* = 3). Metabotropic glutamate receptor antagonists significantly enhanced the inhibition of LLCs in strongly activated MCs, which is mediated by ionotropic glutamate receptor antagonists.

### 3.7. The Development or Enhancement of LLCs by Gabazine Involved Cytoplasmic Ca^2+^ in MCs

**Figure 11:**
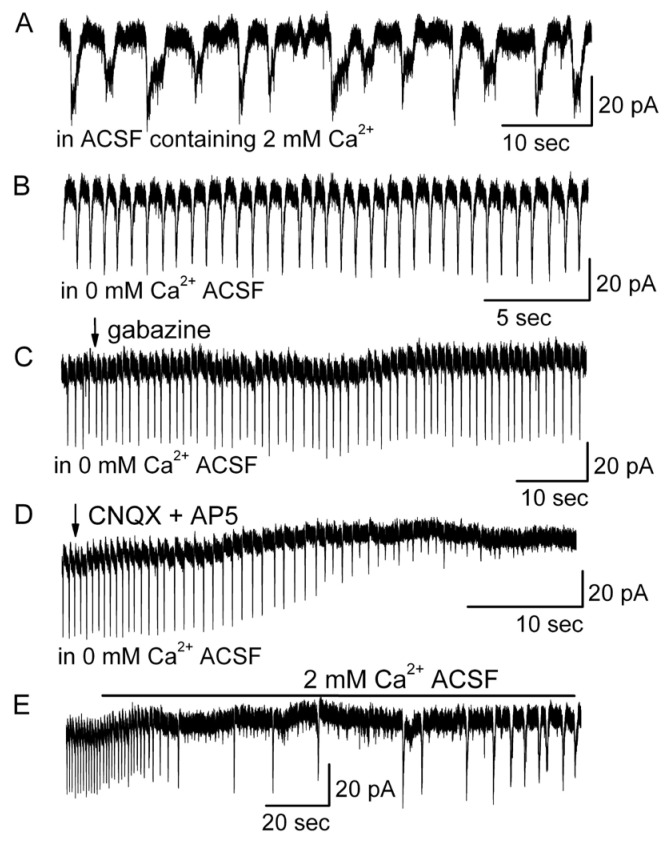
Ca^2+^ regulated LLCs in MCs. (**A**) HP = −60 mV, in ACSF containing 2 mM Ca^2+^. (**B**) Here, 0 mM Ca^2+^ modulated the LLC pattern into short-lasting currents (SLCs) and greatly increased the frequency of the SLCs. (**C**) Gabazine (5 μM) failed to enhance SLC in conditions where ACSF contained 0 mM Ca^2+^ ACSF; HP = −60 mV. (**D**) SLC was blocked by 10 μM CNQX + 50 μM AP5. (**E**) LLCs were recovered by perfusion with ACSF containing 2 mM Ca^2+^. (**A**–**E**) were from the same representative mitral cell.

To understand the mechanisms underlying the transformation of firing patterns or development of LLCs in MCs, the role of Ca^2+^ in these processes was examined. Reducing extracellular [Ca^2+^] is known to decrease cytoplasmic [Ca^2+^] [51,52], thereby impairing the spontaneous release of neurotransmitters at inhibitory synapses mediated by voltage-activated Ca^2+^ channels [43]. Decreasing extracellular [Ca^2+^] from 2 mM to 0 mM eliminated the slow phase of LLCs (Figure 11A,B). In conditions of 0 mM [Ca^2+^], gabazine failed to enhance the strength or duration of LLCs (Figure 11C), suggesting the participation of Ca^2+^ entering MCs and/or cytoplasmic Ca^2+^ in the development of LLCs. In conditions of 0 mM Ca^2+^, LLCs were blocked by CNQX plus AP5 (Figure 11D), suggesting that the fast phase of LLCs was mediated by ionotropic glutamate receptors. The influence of [Ca^2+^] on LLCs through extracellular Ca^2+^ and cytoplasmic Ca^2+^ was reversible (Figure 11E).

## 4. Discussion

Here, we present electrophysiological evidence that both intrinsic and dendrodendritic mechanisms are involved in changing the firing pattern of MCs. MCs, a type of output neuron, exhibit two firing patterns: (a) regular firing of action potentials and (b) long-lasting membrane depolarizations with a burst of action potentials. We found evidence that MCs demonstrated spontaneous regular firing when they lacked synaptic input. This showed that spiking is an intrinsic property in the presence of fast synaptic blockers (NMDA, AMPA, and GABA_A_ receptor blockers). The generation of spontaneous burst firing required a glutamate spillover of the MCs. This idea was supported by the transformation of firing patterns from bursting to regular firing mediated by ionotropic glutamate receptor antagonists. The disinhibition of MCs through blockade of GABA_A_ receptors depolarized the cells to elicit more glutamate released from apical dendrites of MCs that induced the transformation of the firing pattern from regular firing to burst firing. MCs in the MOB form output channels to higher-order olfactory structures by encoding odor information in the form of action potentials [6]. Our results suggest that intrinsic properties of MCs could be a platform on which odor information is encoded through intrinsic and dendrodendritic mechanisms, which are initially processed in glomeruli.

### 4.1. Firing Patterns of Mitral Cells

The firing patterns of MCs reported in the literature are not consistent, and researchers might be confused about the firing patterns of MCs in slice preparations. MCs could express firing as either regular firing or burst firing, and some regular firing could be automatically switched to burst firing.

In slice preparations in which glutamate receptor-mediated input from olfactory bulb nerves is absent, most MCs display spontaneous regular single spikes with more a modest firing frequency (1–6 Hz) [20,44]. Also, spontaneous rhythmic burst firing with lower burst frequency (0.2–0.6 bursts/s) was reported by other groups [34,35,37]. In our recording condition, MCs spontaneously generated two distinct firing patterns: (a) regular firing that discharges single action potentials continuously, and (b) burst firing that discharges bursts of action potentials. Some MCs with spontaneous regular firing pattern automatically transformed into burst firing. In addition, about ~5% of MCs did not generate any action potentials. These were excluded from our analysis because their dendrites might have been truncated during slice preparation.

Due to the lack of sensory input from the olfactory nerve to MCs in slice preparations, the firing pattern of MCs in vitro appears to be different from that in vivo. In vivo, the sampling rhythm in freely breathing preparations is faithfully translated into a strong, synaptically driven subthreshold oscillation in MCs [53,54]. In vitro, instead of the subthreshold oscillation, MCs displayed intrinsic regular firing because of the lack of sensory input from the olfactory nerve to MCs. In vivo, odorants evoke an excitatory response that is characterized by synaptic depolarization as a function of the respiratory cycle with superimposed bursts of action potentials [8,9,10]. In vitro, in response to electrical stimulation of the olfactory nerve [21] or in the presence of GABA_A_ receptor antagonist, MCs also displayed such depolarizations with superimposed bursts of action potentials.

As the integrative firing behaviors encode important odor information, we hypothesize that the distinct firing patterns represent different mechanisms of encoding information that is sent to higher level olfactory centers.

### 4.2. Transformable Firing Patterns in Mitral Cells

In our recording condition, approximately 5–9% of the spontaneous regular firing of MCs automatically transformed into burst firing. Also, we found that burst firing diminished with fewer bursts or it was completely transformed into a regular firing pattern when the cells were injected with a negative current that made the membrane potential more negative. Our results supported the idea that the spontaneously regular firing pattern of MCs persists in the presence of fast synaptic blockers. However, in spontaneous burst firing MCs, fast synaptic blockers preserved the firing of MCs but altered the burst firing pattern into much more regular firing pattern. Until now, very little was known about the transformation of MC firing and the presence of the two spontaneously generated, distinct firing patterns in the same slice or even in the same cell.

Carlson et al. reported that GABA_A_ receptor antagonist gabazine significantly increased the amplitude of LLDs [48]. This may reflect suppression of tonic GABAergic inhibition in the MOB [55,56]. Our results support the idea that disinhibition of the cells through blockade of GABA_A_ receptors tonically excited the cells, as was seen with a gradually increased inward current (Figure 8). It turned out that more glutamate was released from MCs, and LLCs were finally induced or largely enhanced (Figure 8). Gabazine was used to block both tonic GABA_A_ receptors and the inhibitory GABAergic synaptic input. We propose that the firing patterns were transformable due to depolarization of MCs evoked by transmitters. The depolarization of MCs resulted in the spontaneous release of glutamate from their dendritic terminals which transformed the firing pattern in the same cells.

It was thought that MC spontaneous firing is intrinsically generated because it persists in the presence of fast synaptic blockers, which include ionotropic glutamate and GABA_A_ receptor antagonists [21,44]. These results seem to suggest that fast synaptic transmission is not necessary for MC firing. This implies that the MC firing pattern is independent of fast synaptic transmission. If this is true, then synaptic transmission may not be involved in encoding olfactory information. However, odorant stimuli perceived in the nose rely on synaptic transmission. Therefore, it is hard to image how synaptic transmission would not critically participate in encoding odor information. We found that the firing pattern in MCs was transformable, which suggests that it is a relevant feature for encoding odor information.

### 4.3. Burst Firing and LLCs Did Not Show an All-or-None Property

Olfactory nerve-evoked long-lasting depolarizations (LLDs) were thought to be generated in an all-or-none manner because the amplitudes of evoked LLDs were similar to the amplitudes of spontaneous LLDs [48]. If so, the firing pattern of MCs and LLCs should also be generated in an all-or-none manner. However, our results indicated that the generation of burst firing and LLCs did not display an all-or-none property.

We recorded two distinct MC firing patterns: regular firing and burst firing. Among the MCs with regular firing, about 5–9% of them spontaneously switched their firing pattern to burst firing, suggesting that the firing pattern is generated in an all-or-none manner. However, the strength of spontaneously generated burst firing was increased by blocking GABA_A_ receptors (Figure 1B and Figure 3C), i.e., it was possible to modulate the strength of burst firing. Also, the LLCs in MCs were modulated both in amplitude and burst firing rate (Figure 8). These results further indicate that neither burst firing nor the generation of LLCs showed the all-or-none property.

The observation that burst firing and LLCs were not generated in an all-or-none manner has a physiological relevance for MC signaling. Our results indicate that the firing and the firing patterns in MCs are transformable and modifiable. The modulation of firing by neurotransmitters implies that both regular firing and burst firing could be used to encode odorant signal information. At present, it is not known how important the transformation of firing patterns is for encoding olfactory information, and how bursting events, the rate of burst firing, and the latency of bursting participate in olfactory information processing.

### 4.4. Dendrodendritic Modulation as an Added Mechanism for Odor Discrimination Together with Basic Intrinsic Properties for Regular Firing Patterns

Action potential firing and the timing of firing in MCs are thought to encode odor information [57,58] and to play crucial roles in transmitting odor information to higher-order olfactory centers and in the discrimination of odors. The control or modulation of MC firing and firing patterns impacts all subsequent odor coding in the olfactory system. The action potentials of single MCs may carry sufficient information to discriminate odors. The change in firing patterns in terms of rate and timing of action potentials would encode specific information that would be transferred to higher-order neurons. How is odor information encoded to discriminate odorants? GABAergic inputs have been thought to be involved in tuning bursting [59]. Unlike the burst firing in external tufted cells that is intrinsic and cannot be reversed by blocking glutamatergic transmission [22,60], the generation of burst firing in MCs suggests the involvement of glutamatergic synaptic transmission. Within the MOB, dendrodendritic synapses are thought to sharpen patterns of odorant representation among MCs [61]. Our results indicate that dendrodendritic synapses among MCs changed the firing patterns through either depolarization of MCs or by blocking inhibitory GABAergic or excitatory ionotropic glutamatergic transmission. Since the excitatory synaptic input to MCs is confined to the glomerulus, it is likely that intra-glomerular excitation between the tufts of MCs generates or enhances synchronization [40,43] that drives the firing pattern change. Thus, the action potentials and firing patterns were most likely modified dendrodendritically by intra-glomerular excitation between the tufts of MCs, which would contribute to encoding of odor information and odorant discrimination.

What is the function of MC intrinsic properties that generate spontaneous regular firing of MCs? Our results indicated that regular firing can spontaneously transform into burst firing in MCs or be transformed by a blockade of GABA_A_ receptors. The transformed firing patterns were reversed by a blockade of ionotropic glutamate receptors (Figure 3). Our results are supported by in vivo results [19]. In vivo, the rhythmic membrane potential oscillations and bursts are blocked by glutamate receptor antagonists, demonstrating that they are synaptically mediated. The in vivo and in vitro results indicate that the regular firing of MCs reveals not only the intrinsic properties of MCs but also that a basic firing pattern can be modulated or transformed into another firing pattern. Thus, the intrinsic regular firing can be viewed as a basic platform for encoding odor information. In vivo, odorant stimulation evokes bursting [19,62] which suggests that burst firing is just one mechanism to encode information. MCs in vivo generate mostly individual action potentials or none when there is no odorant stimulation. Actually, the individual action potentials or no firing was recorded at resting status or during naris closure [63,64]. Therefore, we propose that the intrinsic membrane properties of MCs serve as a basic platform for encoding odor information in vivo.

## Data Availability

The data presented in this study are available on request from the corresponding author.
